# High-Fat Diet Alters Immunogenic Properties of Circulating and Adipose Tissue-Associated Myeloid-Derived CD45^+^DDR2^+^ Cells

**DOI:** 10.1155/2019/1648614

**Published:** 2019-02-28

**Authors:** Sara J. Sidles, Ying Xiong, M. Rita I. Young, Amanda C. LaRue

**Affiliations:** ^1^Research Services, Ralph H. Johnson VA Medical Center, Charleston, SC 29401, USA; ^2^Department of Pathology and Laboratory Medicine, Medical University of South Carolina, Charleston, SC 29403, USA; ^3^Department of Otolaryngology, Medical University of South Carolina, Charleston, SC 29403, USA

## Abstract

Chronic inflammation is evident in the adipose tissue and periphery of patients with obesity, as well as mouse models of obesity. T cell subsets in obese adipose tissue are skewed towards Th1- and Th17-associated phenotypes and their secreted cytokines contribute to obesity-associated inflammation. Our lab recently identified a novel, myeloid-derived CD45^+^DDR2^+^ cell subset that modulates T cell activity. The current study sought to determine how these myeloid-derived CD45^+^DDR2^+^ cells are altered in the adipose tissue and peripheral blood of preobese mice and how this population modulates T cell activity. C57BL/6 mice were fed with a diet high in milkfat (60%·kcal, HFD) *ad libitum* until a 20% increase in total body weight was reached, and myeloid-derived CD45^+^DDR2^+^ cells and CD4^+^ T cells in visceral adipose tissue (VAT), mammary gland-associated adipose tissue (MGAT), and peripheral blood (PB) were phenotypically analyzed. Also analyzed was whether mediators from MGAT-primed myeloid-derived CD45^+^DDR2^+^ cells stimulate normal CD4^+^ T cell cytokine production. A higher percentage of myeloid-derived CD45^+^DDR2^+^ cells expressed the activation markers MHC II and CD80 in both VAT and MGAT of preobese mice. CD4^+^ T cells were preferentially skewed towards Th1- and Th17-associated phenotypes in the adipose tissue and periphery of preobese mice. *In vitro*, MGAT from HFD-fed mice triggered myeloid-derived CD45^+^DDR2^+^ cells to induce CD4^+^ T cell IFN-*γ* and TNF-*α* production. Taken together, this study shows that myeloid-derived CD45^+^DDR2^+^ cells express markers of immune activation and suggests that they play an immune modulatory role in the adipose tissue of preobese mice.

## 1. Introduction

Obesity is a complex disease that contributes to the development of type 2 diabetes (T2D), cardiovascular disease, and various cancers [1–6]. An increase of 5 kg/m^2^ in body mass index is associated with a 30% increase in all-cause mortality [4]. The pathology of obesity is multifold and includes aberrant insulin growth factor/insulin signaling, altered steroid production, and chronic systemic and local inflammation [4, 6]. However, the full view of immune dysfunction in obesity is unclear.

Mouse models of high-fat diet- (HFD-) induced obesity are typically characterized by at least a 30% increase in total body weight and closely mimic human disease [7–9]. C57BL/6 mice fed with a HFD *ad libitum* for 16-20 weeks exhibit adipocyte hyperplasia, increased fat mass, hypertension, and impaired glucose sensitivity leading to T2D [7, 10, 11]. Overall, less is known about the molecular and immune changes that occur before obesity is fully established. There is some evidence to suggest that short-term HFD feeding in mice results in hyperglycemia and changes in NK T cell and macrophage populations [12, 13]. The current study is focused on the inflammatory changes that occur in the adipose tissue of HFD-fed preobese mice, which are characterized by a 20% increase in total body weight and more closely represent an overweight, or preobese condition vs. obese condition [14].

In obesity, hypertrophied adipose tissue is comprised of a myriad of cell types, including adipocytes, preadipocytes, fibroblasts, and infiltrating immune cells. Previous studies have shown that monocyte-derived macrophages comprise a significant population in obese adipose tissue, where they become classically activated and skewed towards a proinflammatory, “M1” phenotype [15, 16]. Obese adipose tissue-associated F4/80^+^CD11c^+^ “M1” macrophages produce inflammatory cytokines such as interleukin- (IL-) 12 and tumor necrosis factor- (TNF-) *α* and elicit the abnormal production of adipokines/cytokines such as leptin and IL-6 from surrounding adipocytes [15, 17–23]. This cycle of inflammation becomes self-sustaining and, over time, contributes to the reduced insulin sensitivity and metabolic dysfunction observed in patients with obesity and mouse models of obesity [24–27]. In addition to classically activated “M1” macrophages, populations of F4/80^+^CD11c^−^CD206^−^ “M0” macrophages and alternatively activated F4/80^+^CD11c^−^CD206^+^ “M2” macrophages have also been observed in obese adipose tissue, suggesting that the macrophage phenotype is highly heterogeneous [22, 28, 29]. Interestingly, in patients with obesity, adipose tissue is characterized by a large population of CD11c^+^CD206^+^ “M2”-like macrophages, which retain their remodeling capacity but also secrete proinflammatory cytokines and have been associated with insulin resistance [30]. Accumulating evidence suggests that the skewing of monocyte-derived macrophages in obese adipose tissue is a highly complex and diverse process that depends on a number of factors, including the stroma and metabolic signature (i.e., fatty acid accumulation) of the specific adipose depot, as well as the severity of obesity [22, 31, 32].

There is a growing appreciation for the role of T cells in the obese adipose tissue environment. Adipocytes and other stromal cell subsets in obese adipose tissue secrete proinflammatory mediators (e.g., IL-6, MCP-1) that directly activate and skew T cells, even before a dramatic increase in mature tissue macrophages is observed [17, 33–36]. Resultant production of interferon- (IFN-) *γ*, IL-17A, and IL-6 by adipose tissue-activated T cells impacts adipocytes, muscle cells, and hepatocytes by disrupting glucose and lipid homeostasis and contributing to insulin resistance [37–43]. In mice with HFD-induced obesity, increased T cells are evident in the adipose tissue and have been linked to abnormal glucose homeostasis [8, 37, 44, 45]. However, there remains a critical gap in knowledge of how CD4^+^ T cells become skewed towards Th1 vs. Th17 subsets in adipose tissue and how this changes as obesity is established.

Our lab has previously identified a novel population of hematopoietic stem cell- (HSC-) derived discoidin domain receptor 2- (DDR2-) expressing cells called circulating fibroblast precursors (CFPs), defined by their expression of CD45 and DDR2. DDR2, a tyrosine kinase receptor that binds collagens I and III, is known for its role in cell migration and extracellular matrix sensing [46]. There is some evidence to suggest that DDR2 may also play a role in immune cell activation; its expression on a subset of myeloid-derived immune cells was shown to mediate activation and cytokine production [47]. Previous studies in our lab have shown that CD45^+^DDR2^+^ cells are a circulating progenitor population that home to normal tissue as well as pathological environments, including inflammatory tissue and tumor microenvironments [48–50]. Depending on the local milieu, CD45^+^DDR2^+^ cells can differentiate into a spectrum of cell types, including fibroblasts and immune cells [48–50]. While the fibroblastic phenotype of these cells has been studied, considerably less is known about the immunogenic potential of these cells. Recently, we showed that myeloid-derived CD45^+^DDR2^+^ cells isolated from lung tissue of mice with pulmonary fibrosis expressed high levels of CD80 and MHC II and were capable of stimulating an inflammatory T cell cytokine response [49]. This suggests that a subset of myeloid-derived CD45^+^DDR2^+^ cells may play a role in regulating the immune response in inflammatory tissue.

Given that myeloid-derived CD45^+^DDR2^+^ cells home to sites of inflammation and contribute to both myeloid and stromal populations, the present study sought to determine how HFD impacts on the contribution of myeloid-derived CD45^+^DDR2^+^ cells to adipose tissue and their role as immune modulators in the adipose tissue environment of HFD-fed mice. Specifically, we sought to determine how this cell population may be modulating adipose tissue-associated inflammation in a preobese setting, when immune-based therapies may be more effective. To model preobesity, C57BL/6 mice were fed with a milkfat-based HFD or normal diet (ND) *ad libitum* for 8-10 weeks, until HFD-fed mice reached a 20% increase in total body weight compared to ND-fed mice. Myeloid-derived CD45^+^DDR2^+^ cells and CD4^+^ T cells from peripheral blood (PB), mammary gland-associated adipose tissue (MGAT), and visceral adipose tissue (VAT) were analyzed by flow cytometric analysis. We discovered that myeloid-derived CD45^+^DDR2^+^ cells were more activated in the adipose tissue of HFD-fed preobese mice, characterized by increased expression of MHC II and CD80. CD4^+^ T cells in the adipose tissue of HFD-fed mice were preferentially skewed towards proinflammatory Th1- and Th17-type T cells. CD45^+^DDR2^+^ cells cultured in media conditioned by adipose tissue from HFD-fed mice elicited increased production of IFN-*γ* and TNF-*α* by CD4^+^ T cells, suggesting that HSC-derived CD45^+^DDR2^+^ cells play a role in modulating T cell skewing in the adipose tissue environment of HFD-fed mice.

## 2. Materials and Methods

### 2.1. Mouse Model of HFD-Induced Preobesity

Six-week-old female C57BL/6 mice (B6.SJL-Ptprc^a^Pepc^b^/BoyJ, Jackson Laboratories, Bar Harbor, ME) were fed with a HFD (60.3%·kcal from milkfat) or normal diet (ND, 18%·kcal from fat) for 8-10 weeks, until HFD-fed mice exhibited a 20% increase in total body weight and were characterized as preobese (Envigo Teklad Diets, Madison, WI; Table S1). Body weight was recorded weekly. At endpoint, mice were euthanized and peripheral blood (PB) and mammary gland-associated adipose tissue (MGAT) and visceral adipose tissue (VAT) were collected. MGAT consisted of the subcutaneous adipose tissue surrounding the 4th mammary glands. Visceral adipose tissue (VAT) consisted of the adipose tissue in the gonadal region [51]. Mice were maintained at the Animal Research Facility of the Veterans Affairs Medical Center. The study was carried out in accordance with the principles of the Basel Declaration and was reviewed and approved by the Institutional Animal Care and Use Committee at the Ralph H. Johnson VA Medical Center.

### 2.2. Cell and Tissue Culture Medium

For adipose cell and tissue culture, Dulbecco's modified Eagle's medium nutrient mixture F-12 (DMEM-F12, Life Technologies, Grand Island, NY) containing 10% fetal bovine serum (FBS, Atlanta Biologicals, Flowery Branch, GA) was used. For CFP and CD4^+^ T cell coculture, DMEM containing 10% FBS was used.

### 2.3. Spleen Processing and Isolation of CD4^+^ T Cells

The spleens were harvested from 12-16-week-old control female C57BL/6 mice and homogenized in Hank's buffered saline solution (HBSS). Cells were passed through a 70 *μ*m cell strainer and rinsed with HBSS and red blood cells were lysed (ACK Lysing Buffer; Lonza, Walkersville, MD, USA; 3 min). Splenocytes were washed twice with HBSS. CD4^+^ T cells were isolated using a magnetic-based negative selection kit per manufacturer's instructions (Miltenyi Biotec Inc., Auburn, CA). Isolation purity of CD4^+^ T cells based on flow cytometric analysis was 97.37%+0.24.

### 2.4. Blood and Adipose Tissue Processing for Flow Cytometric Analysis

Blood was incubated with PharmLyse (15 min, room temperature) and remaining white blood cells were washed 3X in phosphate-buffered saline (PBS). VAT and MGAT were dissected, finely minced into 1 mm pieces, and digested in PBS containing 1% bovine serum albumin (BSA) with 2 mg/ml type I collagenase (Sigma, St. Louis, Missouri) on a rotary shaker (100 rpm, 1 h, 37°C). Cells were filtered (40 *μ*m), washed (10% FBS/DMEM-F12), and centrifuged (10,000 rpm, 5 min). The upper layer was decanted and the remaining stromal vascular fraction was washed in PBS, incubated with PharmLyse (10 min, room temperature), and washed in PBS before analysis.

### 2.5. Adipose Tissue Explant Culture

Dissected, minced VAT and MGAT were cultured in 10% FBS/DMEM-F12 media in inverted 25 mm^2^ cell culture flasks using a modified organ culture method (37°C, 5% CO_2_, 48 h) [52]. After 48 h of culture, fresh media was added and cells were cultured an additional 48 h. Supernatants were then collected for cytokine analysis and coculture experiments.

### 2.6. Flow Cytometric Analysis of CD45^+^DDR2^+^ Cells and T Cells

Antibodies and reagents used for flow cytometric analysis were from BD Biosciences, unless noted. Cells (1 × 10^6^cells/tube) were washed, resuspended in 1 ml PBS with near-IR dead cell stain (1 : 1000, Invitrogen), and incubated (30 min, 4°C). After washing, cells were resuspended in 10 *μ*l Fc block, containing anti-CD16/CD32 antibody in PBS (1 : 100, 10 min, 4°C). For CD45^+^DDR2^+^ cell analysis, cells were incubated (20 minutes, 4°C) with equal concentrations of the following antibodies or isotype controls: PE-CD45 (Clone 30-F11), APC-DDR2 (N-20, Santa Cruz Biotechnology, Dallas, TX), FITC-CD11b (M1/70), BV421-F4/80 (T45-2342), BV711-CD80 (16-10A1), and PE-Cy7-MHC II (M5/114.15.2, eBioscience, San Diego, CA). Cells were washed and resuspended in 250 *μ*l stain buffer for analysis. For T cell analysis, cells were incubated with 1X cell stimulation cocktail (eBioscience, 5 h, 37°C). Golgi stop was added at the recommended dilution after 1 h of stimulation. Cells were processed as above and stained with equal concentrations of the following antibodies or isotype controls: FITC-CD3e (145-2C11) and APC-CD4 (RM4-5). After washing, cells were resuspended in 1 ml cold Cytofix and incubated (20 min, 4°C). Cells were washed, resuspended in 50 *μ*l Perm/Wash Buffer, and incubated (30 min, room temperature) with equal concentrations of the following antibodies or isotype controls: BV421-IL-17A (TC11-18H10) and PE-Cy7-IFN-*γ* (XMG1.2). Cells were washed in Perm/Wash Buffer and resuspended in 400 *μ*l stain buffer for analysis. Analysis was conducted using a BD Fortessa X-20 flow cytometer.

### 2.7. Coculture of CD45^+^DDR2^+^ Cells and CD4^+^ T Cells

CD45^+^DDR2^+^ cells sorted from the peripheral blood of control mice were cultured at 1 × 10^4^ cells/well in 96-well tissue culture plates precoated with anti-CD3*ε* (1 : 100, Clone 145-2C11, R&D Systems, Minneapolis, MN, USA) in media conditioned by MGAT from HFD- or ND-fed mice (1 : 4, 48 h, 37°C). After 48 h, adipose tissue-conditioned media was replaced with fresh 10% FBS/DMEM. Isolated CD4^+^ T cells from control C57BL/6 mice were added at 1 × 10^5^ cells/well for coculture (72 h, 37°C). Controls consisted of CD4^+^ T cells or CD45^+^DDR2^+^ cells cultured alone in conditioned or fresh media. After 72 h of coculture, selected wells were stimulated with 1X cell stimulation cocktail (eBioscience, 5 h, 37°C). Supernatants were collected for cytokine analysis.

### 2.8. Cytometric Bead Array

The levels of IL-2, IL-4, IL-6, IFN-*γ*, TNF-*α*, IL-17A, and IL-10 were determined using a mouse Th1/Th2/Th17 cytometric bead array kit (BD Biosciences). Levels of MCP-1, GM-CSF, G-CSF, MIP-1*α*, MIP-1*β*, MIG, and RANTES were determined using cytometric bead array flex sets. Relative amounts of each cytokine/chemokine were analyzed using a FACSCanto (BD Biosciences) flow cytometer and FCAP Array Software (Soft Flow Hungary Ltd. for BD Biosciences, St. Louis Park, MN, USA).

### 2.9. Statistical Analyses

Data were reported using the mean as a measure of central tendency ± standard error of the mean. To compare one variable condition between 2 groups, a 2-tailed Student *t-*test was used. To compare one variable condition between 3 or more groups, a one-way ANOVA with Bonferroni correction was used (GraphPad Prism version 5.03). Significance was reported in the 95% confidence interval.

## 3. Results and Discussion

### 3.1. Increased Total Body Weight and Expansion of MGAT and VAT in Female C57BL/6 Mice Fed with a HFD or ND for 8-10 Weeks

**Figure 1:**
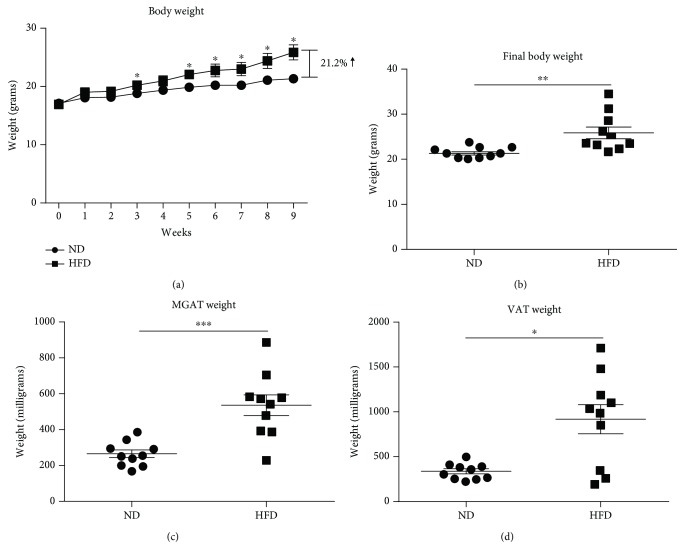
Increased overall body weight and adipose tissue in mice fed with a high-fat diet for 9 weeks compared to lean mice. (a) Total body weight of female C57BL/6 mice fed with a high-fat diet (HFD, 60%·kcal from milkfat) or normal diet (ND) over 9 weeks. (b) Graphical representation of total body weight of HFD- or ND-fed mice at endpoint, when HFD-fed mice reached a ~20% increase in total body weight compared to ND-fed mice. (c, d) Total weight of mammary gland-associated adipose tissue (MGAT) or visceral adipose tissue (VAT) isolated from HFD- or ND-fed mice at endpoint. Data are presented as mean ± SEM of 10 mice per group. ^∗^ *p* < 0.05, ^∗∗^ *p* < 0.01, and ^∗∗∗^ *p* < 0.001.

A diet high in milkfat (60%·kcal) results in consistent weight gain and increased adipose tissue in C57BL/6 mice [8, 11]. Most previous studies have focused on the long-term metabolic and inflammatory impacts of this diet, at 12-16 weeks and beyond, when mice were defined as obese and exhibited at least a 30% increase in total body weight compared to control mice [7–9]. To determine the more immediate impacts of HFD, our study focused on a shorter time point, 8-10 weeks, when mice exhibited a 20% increase in total body weight compared to control mice, to reflect a preobese condition. As shown in Figure 1(a), HFD-fed mice gained more weight than ND-fed mice over 9 weeks. This difference remained statistically significant 5 weeks of postinitiation of diet. At 9 weeks of postinitiation of diet, the mean total body weight of HFD-fed mice was 21.2% greater than that of ND-fed mice (Figure 1(b)), indicating that the preobese condition (20% increase in total body weight) had been met and signifying study endpoint. Examination of adipose tissue showed that HFD-fed, preobese mice were characterized by increased MGAT and VAT by weight at study endpoint (Figures 1(c) and 1(d)).

### 3.2. Increased Percentage of Myeloid-Derived CD45^+^DDR2^+^ Cells Express MHC II and CD80 in MGAT and VAT of Preobese Mice Compared to Lean Mice

Previous studies in our lab have shown that myeloid-derived CD45^+^DDR2^+^ cells home to pathological environments, where they differentiate into fibroblasts and other stromal subsets [48, 50]. Recently, myeloid-derived CD45^+^DDR2^+^ cells were also shown to express markers of immune activation, including CD80 and MHC II, in an inflammatory lung model [49]. These findings led us to examine if a subset of CD45^+^DDR2^+^ cells contributes to inflammation in the adipose tissue of preobese mice. This was accomplished by isolating and phenotyping myeloid-derived CD45^+^DDR2^+^cells from MGAT, VAT, and PB of HFD- vs. ND-fed mice. Both MGAT and VAT were analyzed to provide a more complete view of the phenotype and activation status of CD45^+^DDR2^+^ cells in a local, subcutaneous adipose tissue environment (MGAT) as well as a central, gonadal adipose tissue environment (VAT) [51, 53].

**Figure 2:**
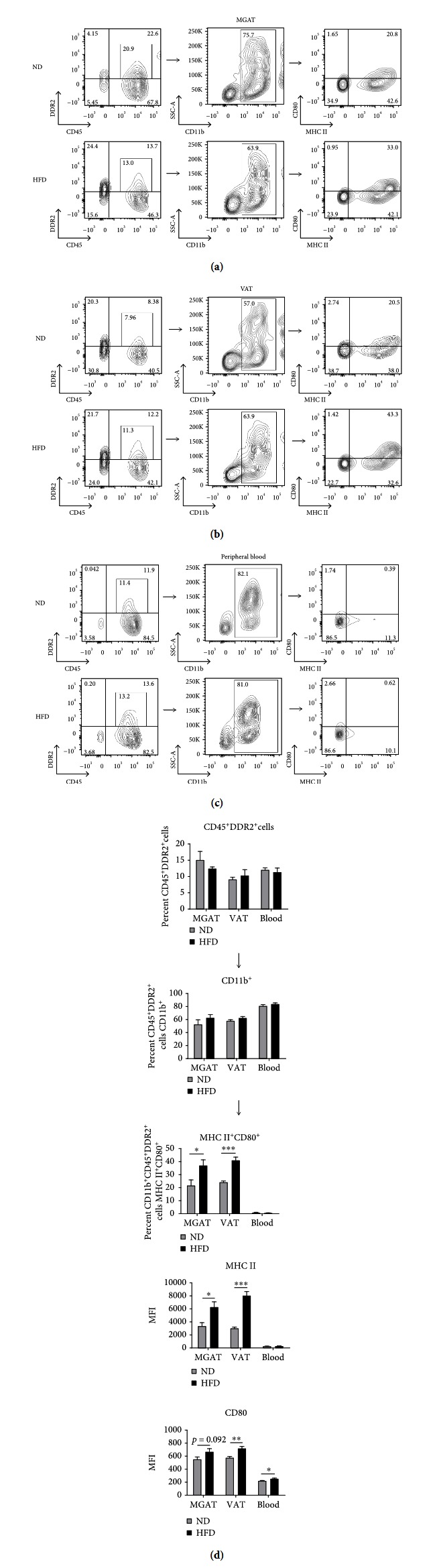
Increased percentage of myeloid-derived CD45^+^DDR2^+^ cells express MHC II and CD80 in mammary gland-associated adipose tissue (MGAT) and visceral adipose tissue (VAT) of preobese mice compared to myeloid-derived CD45^+^DDR2^+^ cells in MGAT and VAT of lean control mice. (a–c) Flow cytometric analysis of a representative mouse from each group, showing MHC II and CD80 expression in CD11b-expressing CD45^+^DDR2^+^ cells from MGAT, VAT, and peripheral blood (PB) of HFD- and ND-fed C57BL/6 mice at endpoint (9 weeks of postinitiation of diet). For flow cytometric analysis, cells were first gated on single, live cell populations. (d) Graphical representation of flow cytometric analysis, showing the percentage of total CD45^+^DDR2^+^ cells, CD11b-expressing CD45^+^DDR2^+^ cells, and MHC II and CD80 expression on CD11b-expressing CD45^+^DDR2^+^ cells in MGAT, VAT, and PB of HFD- vs. ND-fed C57BL/6 mice. Data are presented as mean ± SEM of 5 mice per group. ^∗^ *p* < 0.05, ^∗∗^ *p* < 0.01, and ^∗∗∗^ *p* < 0.001.

Myeloid-derived CD45^+^DDR2^+^ cells were present in both MGAT and VAT, although there was no difference in overall percentage in MGAT (Figures 2(a) and 2(d)), VAT (Figures 2(b) and 2(d)), or PB (Figures 2(c) and 2(d)) between HFD- and ND-fed mice. However, because total fat pad size was increased in HFD-fed mice, we observed an increase in total CD45^+^DDR2^+^ cells in MGAT of HFD-fed mice compared to ND-fed mice (Figure S2). In both MGAT and VAT, a large subset of CD45^+^DDR2^+^ cells expressed CD11b, suggesting that these cells are of the myeloid lineage. The percentage of CD11b-expressing CD45^+^DDR2^+^ cells in MGAT, VAT, or PB was not altered by HFD (Figure 2(d)). However, the total number of CD45^+^DDR2^+^ cells expressing CD11b was increased in MGAT of HFD-fed vs. ND-fed mice (Figure S2). Of note, in PB of both HFD- and ND-fed mice, the majority of CD45^+^DDR2^+^ cells expressed CD11b (Figures 2(c) and 2(d)), suggesting that they represent a progenitor population close to the monocyte lineage.

A higher percentage of myeloid-derived CD45^+^DDR2^+^ cells expressed markers of immune activation in the adipose tissue of HFD-fed mice. In both MGAT and VAT, an increased percentage of myeloid-derived CD45^+^DDR2^+^ cells expressed MHC II and CD80 compared to levels in adipose tissue of ND-fed mice (37.0% vs. 21.6%, ^∗^ *p* = 0.0375 and 41.0% vs. 24.2%, ^∗∗∗^ *p* = 0.0002, Figure 2(d)). The total number of myeloid-derived CD45^+^DDR2^+^ cells expressing MHC II and CD80 in MGAT of HFD-fed was also increased compared to what was observed in ND-fed mice (Figure S2). Analysis of the mean fluorescence intensity (MFI) of activation marker expression showed that MHC II expression on myeloid-derived CD45^+^DDR2^+^ cells was increased in both MGAT and VAT of HFD-fed mice compared to ND-fed mice (^∗^ *p* = 0.0169 and ^∗∗∗^ *p* < 0.001, Figure 2(d)), indicating that MHC II was more highly expressed on this population in the adipose tissue of HFD-fed mice. The MFI of CD80 expression on myeloid-derived CD45^+^DDR2^+^ cells was also increased in MGAT and VAT of HFD-fed mice compared to ND-fed mice, although not statistically significant in MGAT (*p* = 0.0923 and ^∗∗^ *p* = 0.0048, respectively). Overall, these data suggest that the myeloid-derived CD45^+^DDR2^+^ cell population may be skewed towards an activated immune cell phenotype in the adipose tissue of HFD-fed mice, characterized by increased expression of markers of antigen presentation and activation.

There was no statistically significant difference in the percentage of myeloid-derived CD45^+^DDR2^+^ cells expressing MHC II or CD80 in PB of HFD- vs. ND-fed mice, which was not surprising given that CD45^+^DDR2^+^ cells are a circulating progenitor population. However, the MFI of CD80 expression on myeloid-derived CD45^+^DDR2^+^ cells in PB of HFD-fed mice was increased compared to myeloid-derived CD45^+^DDR2^+^ cells in ND-fed mice (^∗^ *p* = 0.0184, Figure 2(d)), suggesting that a subset of these cells may become activated even before reaching adipose tissue.

Analysis of myeloid-derived CD45^+^DDR2^−^ cells in MGAT of HFD-fed mice showed that these cells were not as activated as the DDR2^+^ subset, characterized by significantly lower levels of expression of both MHC II and CD80. In MGAT of HFD-fed mice, the MFI of MHC II expression on myeloid-derived CD45^+^DDR2^−^ cells was significantly lower than that of DDR2^+^ cells (1124 vs. 6279, ^∗∗∗^ *p* = 0.0002) and was not significantly different than what was observed in ND-fed mice (*p* = 0.7766, Figure S3). The MFI of CD80 expression of myeloid-derived CD45^+^DDR2^−^ cells in MGAT of HFD-fed mice was also significantly lower than that of DDR2^+^ cells (382.2 vs. 667.2, ^∗∗∗^ *p* = 0.0005, Figure S3).

As was seen in MGAT, CD45^+^DDR2^−^ cells in VAT and PB of HFD-fed mice were not as highly activated as CD45^+^DDR2^+^ cells. In VAT of HFD-fed mice, the expression of both MHC II and CD80 on CD45^+^DDR2^−^ cells was significantly lower than that of DDR2^+^ cells (1290 vs. 8040, ^∗∗∗^ *p* < 0.0001 and 376.4 vs. 718.4, ^∗∗∗^ *p* < 0.0001). As was observed in MGAT, the levels of MHC II and CD80 expression on CD45^+^DDR2^−^ cells in HFD-fed mice were not significantly different than the levels observed on CD45^+^DDR2^−^ cells in ND-fed mice (*p* = 0.1508 and *p* = 0.2586, Figure S3). In PB of HFD-fed mice, the MFI of both MHC II and CD80 expression on CD45^+^DDR2^−^ cells was lower than what was observed on CD45^+^DDR2^+^ cells (169.4 vs. 280.8, *p* = 0.0653 and 182.8 vs. 254.0, ^∗∗∗^ *p* < 0.0001, Figure S3). Overall, myeloid-derived CD45^+^DDR2^−^ cells were not as highly activated as the CD45^+^DDR2^+^ subset in the adipose tissue or PB, and more importantly, do not exhibit increased activation in the adipose tissue of HFD-fed mice.

Taken together, these data show that myeloid-derived CD45^+^DDR2^+^ cells are a component of adipose tissue that become highly activated in HFD-fed, preobese mice and suggest that this cell subset contributes to the inflammatory adipose tissue environment.

### 3.3. Increased Percentage of T Cells Produce IL-17A in MGAT and PB of Preobese Mice Compared to Lean Mice

Adipose tissue of obese mice is inflammatory and marked by the infiltration of activated immune cells, including macrophages and Th1-type cells [15, 44]. The contribution of other proinflammatory T cell subsets, including Th17 cells, to adipose tissue in obese or preobese mice has not been well defined. To determine how CD4^+^ T cells are skewed in the adipose tissue and periphery of preobese mice, IL-17A and IFN-*γ* expression in T cells isolated from MGAT, VAT, and PB of HFD- vs. ND-fed mice was analyzed.

**Figure 3:**
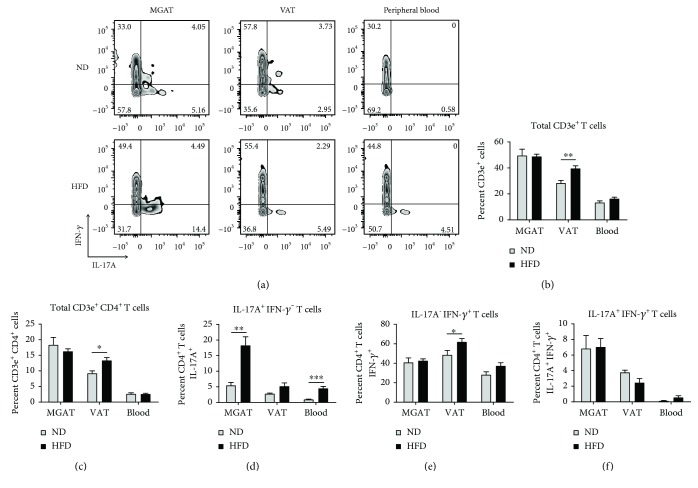
Increased percentage of CD4^+^ T cells express IL-17A in MGAT and blood of preobese mice compared to CD4^+^ T cells in MGAT and blood of lean control mice. (a) Flow cytometric analysis of a representative mouse from each group, showing IL-17A and IFN-*γ* cytokine expression in CD3e^+^CD4^+^ T cell populations from MGAT, visceral adipose tissue (VAT), and peripheral blood (PB) of HFD- and ND-fed C57BL/6 mice at endpoint (9 weeks of postinitiation of diet). Cells were stimulated with PMA/ionomycin cocktail + protein transport inhibitor for 5 h at 37°C prior to fixation, permeabilization, and staining. For flow cytometric analysis of cytokine expression, cells were first gated on single, live, CD3e^+^, and CD4^+^ T cell populations (Figure S4). (b–f) Graphical representation of the percentage of total CD3e^+^ T cells, total CD3e^+^CD4^+^ T cells, IL-17A^+^CD4^+^ T cells, IFN-*γ* ^+^CD4^+^ T cells, and IL-17A^+^IFN-*γ* ^+^CD4^+^ T cells in MGAT, VAT, and PB of HFD- vs. ND-fed mice. Data are presented as mean ± SEM of 5 mice per group. ^∗^ *p* < 0.05, ^∗∗^ *p* < 0.01, and ^∗∗∗^ *p* < 0.001.

The percentage of CD3e^+^ T cells in VAT of HFD-fed mice was increased compared to levels in ND-fed mice (39.6% vs. 28.3%, ^∗∗^ *p* = 0.0049, Figure 3(b)), but this difference was not seen in MGAT or peripheral blood. An increased percentage of total CD3e^+^CD4^+^ T cells was also observed in VAT of HFD-fed mice (^∗^ *p* = 0.0126, Figure 3(c), Figure S4). No difference in the percentage of total CD3e^+^CD4^+^ T cells was observed in MGAT or peripheral blood of HFD-fed vs. ND-fed mice.

Analysis of T cell subsets showed an increased percentage of CD4^+^ T cells expressing IL-17A in MGAT of HFD-fed mice compared to CD4^+^ T cells in MGAT of ND-fed mice (18.3% vs. 5.5%, ^∗∗^ *p* = 0.0023, Figure 3(d)). Although a large percentage of CD4^+^ T cells expressed IFN-*γ* in MGAT, no difference was observed between HFD- and ND-fed mice (Figure 3(e)). A small population of CD4^+^ T cells in MGAT expressed both IL-17A and IFN-*γ*, but no difference was observed between groups (Figure 3(f)).

In VAT of HFD-fed mice, an increased although not statistically significant percentage of CD4^+^ T cells expressed IL-17A compared to ND-fed mice, and the total population of IL-17A-expressing cells was lower compared to levels in MGAT (*p* = 0.0605, Figure 3(d)). VAT of HFD-fed mice was characterized by an increased percentage of IFN-*γ*-expressing CD4^+^ T cells compared to ND-fed mice (62.0% vs. 48.6%, ^∗^ *p* = 0.0483, Figures 3(a) and 3(e)). A small population of CD4^+^ T cells expressed both IL-17A and IFN-*γ* in VAT, but no difference was observed between HFD- and ND-fed mice (Figure 3(f)). In PB, an increased percentage of CD4^+^ T cells expressed IL-17A in HFD- vs. ND-fed mice (^∗∗∗^ *p* < 0.0004, Figure 3(d)). A large proportion of CD4^+^ T cells also expressed Th1-associated IFN-*γ*, although no stastically significant difference between HFD- and ND-fed mice was observed (*p* = 0.0788, Figure 3(e)).

Together, these data show that a greater percentage of CD4^+^ T cells is stimulated in the adipose tissue and PB of HFD-fed preobese mice. In MGAT and PB, the increase in levels of CD4^+^ T cells expressing IL-17A suggests that they are skewed towards a Th17 phenotype. In VAT, the increase in levels of CD4^+^ T cells expressing IFN-*γ* suggests that they are skewed towards a Th1 phenotype.

### 3.4. MGAT from HFD-Fed Mice Triggers CD45^+^DDR2^+^ Cells to Activate T Cell Production of IFN-*γ* and TNF-*α*

Previously, we showed that a subset of activated CD45^+^DDR2^+^ cell shaves the capacity to skew CD4^+^ T cells towards a Th17 phenotype [49]. To determine if adipose tissue from HFD-fed mice triggers CD45^+^DDR2^+^ cells to stimulate T cell cytokine production, sorted CD45^+^DDR2^+^ cells from peripheral blood of control, ND-fed mice were primed in media conditioned by MGAT from HFD- or ND-fed mice and then cultured with CD4^+^ T cells from control, ND-fed mice. Following coculture, Th1, Th2, and Th17 cell-associated cytokines produced by CD4^+^ T cells were analyzed.

**Figure 4:**
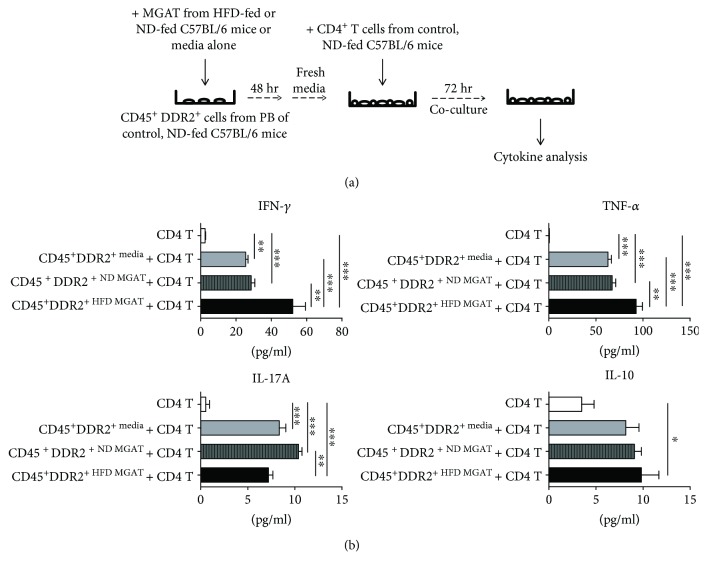
MGAT from HFD-fed mice triggers CD45^+^DDR2^+^ cells to elicit increased production of IFN-*γ* and TNF-*α* from CD4^+^ T cells. (a) Experimental overview: sorted CD45^+^DDR2^+^cells from peripheral blood of control, ND-fed C57BL/6 mice were cultured in media conditioned by MGAT from HFD- or ND-fed mice at 1 × 10^4^ cells/well for 48 h at 37°C. For T cell coculture, media was replaced and splenic CD4^+^ T cells from control, ND-fed C57BL/6 mice were added to CD45^+^DDR2^+^ cell cultures at 1 × 10^5^ cells/well for 72 h. Supernatants were collected and levels of Th1-, Th2-, and Th17-associated cytokines were analyzed by cytometric bead array. (b) Graphical representation of levels of IFN-*γ*, TNF-*α*, IL-17A, and IL-10 detected in supernatants. Data are presented as mean ± SEM of triplicate wells analyzed in duplicate. ^∗^ *p* < 0.05, ^∗∗^ *p* < 0.01, and ^∗∗∗^ *p* < 0.001.

CD45^+^DDR2^+^ cells cultured in media conditioned by MGAT from HFD-fed mice (CD45^+^DDR2^+HFD/MGAT^) stimulated increased production of the Th1-associated cytokine IFN-*γ* from CD4^+^ T cells compared to CD45^+^DDR2^+^ cells cultured in media conditioned by adipose tissue from ND-fed mice (CD45^+^DDR2^+ND/MGAT^, ^∗∗^ *p* < 0.01) or media alone (CD45^+^DDR2^+media^, ^∗∗∗^ *p* < 0.001, Figure 4). CD45^+^DDR2^+HFD/MGAT^ also stimulated increased production of the proinflammatory mediator TNF-*α* from CD4^+^ T cells compared to CD45^+^DDR2^+ND/MGAT^(^∗∗^ *p* < 0.01) or CFP^media^ (^∗∗∗^ *p* < 0.001). These data suggest that CD45^+^DDR2^+HFD/MGAT^ skew T cell cytokine production and promote a Th1-type, inflammatory response.

In contrast, CD45^+^DDR2^+ND/MGAT^ stimulated increased production of the Th17-associated cytokine IL-17A from CD4^+^ T cells compared to CD45^+^DDR2^+HFD/MGAT^ (^∗∗^ *p* < 0.01, Figure 4). However, it is important to note that both CD45^+^DDR2^+HFD/MGAT^ and CD45^+^DDR2^+media^ stimulated increased IL-17A production from CD4^+^ T cells compared to CD4^+^ T cells cultured in the absence of CD45^+^DDR2^+^ cells (^∗∗∗^ *p* < 0.001). Taken together, this data shows that CD45^+^DDR2^+^ cells alone elicit production of inflammatory IL-17A from CD4^+^ T cells, although to a lesser degree than IFN-*γ in vitro*.

Since IL-10 is a known immune inhibitory mediator, its expression was also measured to more completely assess the immune regulatory capacity of CD45^+^DDR2^+^ cells. There were no statistically significant differences in the production of Th2 cell-associated cytokine IL-10 by CD4^+^ T cells cultured with CD45^+^DDR2^+HFD/MGAT^ vs. CD45^+^DDR2^+ND/MGAT^, although CD45^+^DDR2^+HFD/MGAT^ elicited more IL-10 production from CD4^+^ T cells compared to T cells cultured in the absence of CD45^+^DDR2^+^ cells (^∗^ *p* < 0.05). Overall, the levels of IL-10 produced by T cells were significantly lower than the levels of IFN-*γ* and TNF-*α*. Taken together, the data show that CD45^+^DDR2^+^ cells preconditioned in the HFD MGAT environment preferentially skew T cells towards a proinflammatory phenotype.

As a negative control, CD45^+^DDR2^+^ cells were cultured alone in fresh media or media conditioned by adipose tissue from HFD- or ND-fed mice; there were no detectable levels of Th1, Th2, or Th17 cell-associated cytokines in these cultures (data not shown). We found that CD45^+^DDR2^+^ cells primed in media conditioned by VAT from HFD mice stimulated T cell cytokine production of IFN-*γ* and TNF-*α*, but to a lesser degree (~8- and ~2-fold less, respectively, data not shown), suggesting that CD45^+^DDR2^+^ cells differentially modulate T cells in different adipose environments and that these cells may play a more significant proinflammatory role in MGAT of HFD-fed preobese mice. Overall, our data suggest that CD45^+^DDR2^+^ cells exposed to MGAT of HFD-fed mice preferentially skew T cells towards a proinflammatory, Th1-type phenotype.

## 4. Conclusions

To our knowledge, this is the first study examining CD45^+^DDR2^+^ cells in the adipose tissue of HFD-fed mice at the preobese stage and suggests a novel role for myeloid-derived CD45^+^DDR2^+^ cells in modulating the inflammatory immune response in adipose tissue.

The expression of DDR2 on immune cells and the role it plays in immune cell function has not been well defined. DDR2, a tyrosine receptor kinase, binds collagens I and III and is traditionally involved in extracellular matrix sensing and cell migration [46]. Previous studies in human vascular smooth muscle cells and mouse fibroblasts showed that DDR2 regulates expression and/or activation of MMP-1 and MMP-2, respectively [54, 55]. We have previously shown that HSC-derived DDR2^+^ cells home to tumor via the CCR2/MCP-1 axis and are capable of differentiating into fibroblasts in the local tumor environment [48]. However, the immune phenotype of CD45^+^DDR2^+^ cells has not been extensively investigated. One previous study showed that DDR2 is expressed on a subset of dendritic cells and that binding of collagen I to DDR2 leads to increased expression of the activation marker CD86 and increased production of IL-12, a proinflammatory cytokine involved in Th1-type skewing [47]. This suggests that DDR2 expression on a subset of myeloid-derived immune cells may play a role in activation and inflammatory cytokine production in these cells. Our previous work in a mouse model of silica-induced pulmonary fibrosis showed that a subset of myeloid-derived CD45^+^DDR2^+^ cells honed to lung tissue, expressed markers of immune activation, and were capable of skewing T cell cytokine production [49]. These prior studies suggest that myeloid-derived CD45^+^DDR2^+^ cells play a role in modulating the immune response and led us to evaluate their role in promoting inflammation in the preobese adipose tissue environment.

Many models of HFD-induced obesity consider 16-20 weeks of feeding as study endpoint, when mice are characterized as obese and exhibit at least a 30% increase in total body weight [7–9, 11]. Our study is focused on immune changes that are occurring much earlier in the progression to obesity—what we are characterizing as a preobese state—when mice exhibit a 20% increase in total body weight compared to ND-fed mice. As defined in the current study, HFD-fed preobese mice may more closely mirror the overweight condition in human patients [14] and may also offer an ideal model to study immune-based therapies. In the current study, C57BL/6 mice were fed with a diet high in milkfat (60.3% kcal from fat, Table S1), as this diet has been shown to induce metabolic, cardiac, and inflammatory changes in C57BL/6 mice that closely mirror what is observed in obese patients [56–59]. This milkfat-based diet was shown to induce metabolic and inflammatory changes in C57BL/6 mice at earlier time points than a lard-based diet (8 weeks vs. 20 weeks and beyond), suggesting that it is ideal for the current preobese model [56, 60]. It is important to note that HFD feeding has differential effects on weight gain, metabolism, and inflammation in male vs. female mice [61–63]. However, many of these studies utilized lard- or soybean-based HFDs, and overall less is known about the effects of a milkfat-based HFD. We chose to focus on female mice in the current study to provide a baseline for future work examining the role of myeloid-derived CD45^+^DDR2^+^ cells in obesity-associated breast cancer. We have previously observed changes in circulating IL-6 levels and insulin and glucose metabolism in female C57BL/6 mice following 12 weeks of milkfat-based HFD feeding, although to a lesser extent than what was observed in male mice (data not published). The effects of a milkfat-based HFD on CD45^+^DDR2^+^ cell activation and T cell skewing at the preobese state (after ~8 weeks of feeding) have not been previously described and are the focus of the current study.

In our preobese model, CD45^+^DDR2^+^ cells and T cells were examined in two adipose tissue regions: VAT and MGAT. Previous reports have shown that VAT from the gonadal region of obese mice, which often represents the largest and most accessible fat pad, is characterized by elevated oxidative stress, altered glucose metabolism, and increased expression of inflammatory markers [53, 64–67]. There is growing evidence to suggest that MGAT in overweight or obese patients is characterized by increased levels of inflammatory immune cells and adipocytes, which have been shown to contribute to the development and progression of breast cancer [68–72]. Given that considerably less is known about the immune alterations that occur in subcutaneous MGAT in HFD-fed preobese mice, the current study sought to address this question.

In control mice, we found that CD45^+^DDR2^+^cells are present in both MGAT and VAT. While the overall percentage of myeloid-derived CD45^+^DDR2^+^ cells in adipose tissue was unchanged in HFD-fed preobese mice, a significantly higher percentage of myeloid-derived CD45^+^DDR2^+^ cells expressed MHC II and CD80 in both MGAT and VAT. Increased expression of MHC II, which presents antigen/peptide to a T cell via the T cell receptor, and CD80, a costimulatory molecule that binds to CD28 on an interacting T cell, are characteristics of an activated antigen-presenting cell [73–75]. In the adipose tissue of HFD-fed preobese mice, myeloid-derived CD45^+^DDR2^+^ cells were characterized by significantly increased expression of both MHC II and CD80 compared to myeloid-derived CD45^+^DDR2^+^ cells in the adipose tissue of ND-fed mice, indicating that these cells were more activated in the HFD-fed adipose environment.

Changes in the activation of myeloid-derived CD45^+^DDR2^+^ cells were also observed in PB of HFD-fed mice, although to a lesser extent than what was observed in adipose tissue. Previous studies have shown that CD45^+^DDR2^+^ cells differentiate into activated fibroblasts and immune cells in inflammatory tissue environments (i.e., tumor, inflammatory lung) [48–50]. In the current study, the intensity of CD80 expression was increased on myeloid-derived CD45^+^DDR2^+^ cells in the peripheral blood of HFD-fed mice, demonstrating that a subset of this population expresses markers of activation even before they reach the adipose tissue. Thus, at least in a model of preobesity, the activation status of myeloid-derived CD45^+^DDR2^+^ cells may be a useful biomarker for immune activation in the progression to obesity. Further, as they are a circulating progenitor population, CD45^+^DDR2^+^ cells may represent an important target for therapies aimed at reducing inflammation by redirecting their skewing away from an activated immune state.

Analysis of myeloid-derived CD45^+^DDR2^−^ cells in the adipose tissue of HFD-fed mice showed that these cells were not as highly activated as the DDR2^+^ population; furthermore, the extent of activation of DDR2^−^ cells, as defined by the MFI of MHC II and CD80 expression, was not altered in HFD-fed mice vs. ND-fed mice (Figure S3). Taken together, our data show that myeloid-derived CD45^+^DDR2^+^ cells are highly activated in HFD-fed preobese mice and suggest that a subset of these cells may be modulating the inflammatory immune response before obesity is established.

Future studies will focus on defining the differentiation potential of myeloid-derived CD45^+^DDR2^+^ cells in the adipose tissue of HFD-fed preobese mice, using the expression of macrophage, dendritic cell, and fibroblast markers. It will be important to compare the progenitor CD45^+^DDR2^+^ cells presented herein to traditional monocyte-derived macrophages in the adipose tissue and blood of preobese vs. control mice, based on expression of specific macrophage markers such as F4/80, CD11c, CD64, CD206, and Ly6c. Previous studies have shown that the skewing of monocyte-derived macrophages in obese adipose tissue is a highly diverse process and results in the differentiation of both F4/80^+^CD11c^+^ “M1”-like macrophages as well as F4/80^+^CD11c^−^CD206^+^ “M2”-like macrophages [15, 22, 28, 29]. Previous studies in our lab have shown that a subset of myeloid-derived CD45^+^DDR2^+^ cells migrates in response to monocyte-derived chemoattractant protein- (MCP-) 1 [48] and express CD11c in inflammatory lung tissue [49]. It will be important to determine if the myeloid-derived CD45^+^DDR2^+^ cells presented herein contribute to “M1” or “M2” macrophage populations, or a unique macrophage population in adipose tissue, and how this changes as obesity is established. Preliminary analysis of F4/80 expression on myeloid-derived CD45^+^DDR2^+^ cells in MGAT of HFD-fed, preobese mice has shown ~61% of this population expresses F4/80 and was not statistically different than what was observed in ND-fed mice (data not shown). Together, these data suggest that a subset of CD45^+^DDR2^+^ cells differentiates into macrophages or macrophage-like cells in tissue and may represent a progenitor population from which activated tissue macrophages arise. Further defining how CD45^+^DDR2^+^ cells differentiate into immune cell subsets in inflammatory tissue will help guide future studies aimed at targeting this circulating progenitor population.

T cell skewing in adipose tissue contributes to inflammation and may also impact obesity-related metabolic dysfunction. In patients with obesity, adipose tissue-associated and circulating T cells are preferentially skewed towards inflammatory Th1- and/or Th17-type T cells and studies have shown that IFN-*γ* and IL-17A contribute to aberrant glucose and lipid homeostasis in several cell types, including adipocytes [38–40, 76]. In the current study, we show that changes in total T cells and T cell skewing are evident in HFD-fed preobese mice, which are characterized by a 20% increase in total body weight. In both MGAT and PB of HFD-fed mice, the increase in CD4^+^ T cells expressing IL-17A suggests skewing towards a proinflammatory Th17 phenotype. In MGAT of HFD-fed mice, we found 3.4X IL-17A-expressing CD4^+^ T cells compared to MGAT of ND-fed mice, paralleling what is observed in the subcutaneous adipose tissue of patients with obesity-associated T2D [39]. To our knowledge, this is the first report showing increased IL-17A-expressing cells in MGAT of HFD-fed preobese mice and suggests that our model may be a clinically relevant tool for investigating immune alterations associated with the overweight, or preobese, state. In VAT of preobese mice, we observed an increase in CD4^+^ T cells expressing IFN-*γ*, suggesting a skewing towards a Th1 phenotype. This observation supports previous studies which showed a preferential skewing of CD4^+^ T cells towards Tbet-expressing Th1-type cells in VAT of patients with obesity and HFD-induced mouse models of obesity [36, 43, 77]. Of note, an IL-17A^+^IFN-*γ* ^+^ T cell population was observed in both MGAT and VAT and may represent a T cell in transition from a Th17 to Th1 phenotype or a highly activated Th17 cell [78]. Future studies examining the expression of transcription factors such as ROR*γ*t and Tbet in adipose tissue-associated T cells may further delineate these populations. Overall, the data show a differential skewing of T cells in different adipose tissue environments and suggest that HFD promotes both Th1- and Th17-type skewing, even before obesity is established.

While our studies focused on the impact of HFD in female animals, given the sex-specific response to HFD feeding [61–63], it will be important to investigate CD45^+^DDR2^+^ and T cell populations in the peripheral blood and adipose tissue of preobese, male C57BL/6 mice in future studies. There is evidence to suggest that total T cells are also increased in the adipose tissue of HFD-fed, male mice, although reports of how CD4^+^ T cells are skewed in specific adipose depots, particularly at earlier time points, are limited. One study showed that the percentage of total CD3e^+^ T cells was increased in epididymal (i.e., visceral) adipose tissue of HFD-fed, male C57BL/6 mice after 22 weeks of HFD feeding [44]. Increased levels of IFN-*γ* were detected in epididymal adipose tissue isolated from HFD-fed mice after 12 weeks of HFD feeding, suggesting that T cells may be skewed towards a Th1-type phenotype in this adipose depot at an earlier time point [44]. We observed a similar increase in the percentage of total CD3e^+^ T cells and IFN-*γ*-expressing T cells in visceral adipose tissue of HFD-fed, female C57BL/6 mice, though at an earlier time point (8-10 weeks). A few studies in HFD-fed, male C57BL/6 mice have also shown that IL-17A-expressing CD4^+^ T cells are preferentially increased in inguinal (i.e., subcutaneous) adipose tissue compared to ND-fed mice, after 8 weeks of feeding [43]. We observed similar increases in IL-17A-expressing CD4^+^ T cells in MGAT (i.e., subcutaneous) of HFD-fed, female C57BL/6 mice, suggesting that HFD may promote Th17 cell skewing in subcutaneous adipose tissue, independent of sex.

Because we observed a ~3-fold increase in the percentage of inflammatory IL-17A-expressing T cells in MGAT of HFD-fed, preobese mice compared to ND-fed mice, we next sought to hone in on the mechanism by which activated CD45^+^DDR2^+^ cells impact immune activation in this environment. For these studies, MGAT-conditioned media was derived using the organ culture method, to preserve the cellular composition and paracrine interactions within the adipose tissue [52]. In this way, we could examine the impact of the MGAT environment, as a whole, on CD45^+^DDR2^+^ cell-mediated T cell skewing. We found that CD45^+^DDR2^+^ cells from control C57BL/6 mice exposed to MGAT from HFD-fed mice had a greater capacity to stimulate normal CD4^+^ T cell production of IFN-*γ* and TNF-*α*. This suggests that, in the adipose environment of HFD-fed mice, CD45^+^DDR2^+^ cells promote Th1-type skewing. CD45^+^DDR2^+ND/MGAT^ and CD45^+^DDR2^+media^ also induced increased production of IFN-*γ* and TNF-*α*, although to a lesser extent than CD45^+^DDR2^+HFD/MGAT^. These data indicate that a CD45^+^DDR2^+^ cell alone has the capacity to activate a T cell but becomes more activated in the HFD-associated adipose tissue environment, resulting in increased Th1 cell-associated cytokine production. One mechanism by which CD45^+^DDR2^+^ cells may be stimulating inflammatory T cell cytokine production in the HFD environment is via production of proinflammatory mediators such as MIG, MIP-1*α*, and RANTES(Figure S5). Future studies using direct vs. indirect coculture systems will further delineate the mechanism by which CD45^+^DDR2^+^ cells skew T cell cytokine production.

While a stark increase in the percentage of IL-17A-producing CD4^+^ T cells was observed in MGAT of HFD-fed mice compared to CD4^+^ T cells in ND-fed mice, we did not observe a dramatic increase in IL-17A production by CD4^+^ T cells cocultured with CD45^+^DDR2^+^ cells preconditioned in the HFD- vs. ND-fed MGAT environment. These differences could be due to the limitations of the *in vitro* system; the coculture assay includes only preconditioned CD45^+^DDR2^+^ cells and CD4^+^ T cells and we cannot exclude the possibility that CD45^+^DDR2^+^ cells may rely on other cell types in the mammary gland, including preadipocytes, adipocytes, and stromal cells, to elicit Th17 cell skewing. Further, while the percentage of IL-17A-expressing CD4^+^ T cells was increased in MGAT of HFD-fed mice, the level of IL-17A produced by these cells ex vivo was not assessed (precluded by intracellular staining) and therefore we cannot directly compare the results. It is important to note that we observed significantly higher levels of IFN-*γ* production by CD4^+^ T cells compared to IL-17A production *in vitro*. One interpretation of this result is that CD45^+^DDR2^+^ cells preconditioned in the HFD-fed MGAT environment skew CD4^+^ T cells more strongly towards a Th1-type phenotype. It is also possible that a population of CD4^+^T cells producing both IFN-*γ* and IL-17A is induced by coculture with CD45^+^DDR2^+HFD/MGAT^ *in vitro*. These cells may represent a population in transition from a Th1 to Th17 phenotype or a highly activated Th17 cell [78]. This is supported by the *in vivo* data, which showed a population of CD4^+^ T cells expressing both IFN-*γ* and IL-17A in MGAT of HFD- and ND-fed mice.

The level of the Th2 cell-associated, inhibitory mediator IL-10 produced by T cells cultured with CD45^+^DDR2^+HFD/MGAT^ was increased compared to that produced by T cells alone or T cells cultured with CD45^+^DDR2^+ND/MGAT^ or CD45^+^DDR2^+media^. This was a puzzling observation in light of the concurrent Th1 cell skewing and may indicate that CD45^+^DDR2^+^ cells in adipose tissue of HFD-fed mice also promote a Th2-type response, although to a lesser degree. Alternatively, the increased IL-10 production may reflect a compensatory “toning down” of the inflammatory response. Overall, the current study demonstrates, for the first time, that CD45^+^DDR2^+^ cells have the capacity to skew T cell cytokine production in the MGAT environment of HFD-fed mice and points to a novel role for CD45^+^DDR2^+^ cells in promoting inflammation in this adipose depot.

The mechanism by which adipose tissue from HFD-fed mice activates CD45^+^DDR2^+^ cells to stimulate T cell cytokine production remains to be elucidated; one possibility is via the production of inflammatory mediators such as IL-6, MCP-1, G-CSF, MIP-1*α*, and MIP-1*β* which have been shown to be upregulated in the adipose environment of HFD-fed mice and were detected in adipose tissue explant cultures in the current study (data not shown) [17, 33, 34, 36]. Future studies using blocking antibodies to secreted inflammatory mediators will help to define this mechanism *in vitro*. It may also be important to investigate how CD45^+^DDR2^+^ cells preconditioned in the MGAT environment of HFD-fed mice modulate CD4^+^ T cells from HFD- vs. ND-fed mice, as there may be differences in the activation and functional profile of splenic T cells in these mice. *In vivo* manipulation of this population will be an important focus of future studies. Because specific inhibitors of activators of DDR2 are not currently commercially available, these cells are difficult to target *in vivo*. Furthermore, because the CD45^+^DDR2^+^ cell population we have identified is a progenitor population that has the capacity to differentiate into a wide spectrum of cells, including fibroblasts [48, 50] and immune cells [49], the experimental parameters of when to transfer and if/when they home to adipose tissue, in a way that is analogous to their homeostatic arrival, remains to be determined and will be the focus of future studies. While the *in vitro* system used in the current study is a simplified model of the *in vivo* adipose tissue environment, it suggests that CD45^+^DDR2^+^ cells exposed to the HFD-fed adipose environment contribute to T cell activation and provide important insight for future experiments in the preobese mouse model.

Our current understanding of inflammation in preobesity and obesity—and the network of events leading to T cell activation in adipose tissue—is not complete. Recent evidence points to a direct link between Th1 and Th17 cells in the adipose tissue and aberrant glucose homeostasis, suggesting that T cell skewing may play a critical role not only in obesity-related inflammation but also metabolic dysfunction [37–44]. The current study shows, for the first time, that myeloid-derived CD45^+^DDR2^+^ cells express markers of immune activation in the adipose tissue of HFD-fed preobese mice and suggests that they promote Th1-type skewing and the production of inflammatory cytokines. Because myeloid-derived CD45^+^DDR2^+^ cells are a circulating progenitor population, they may represent an important target for therapies aimed at reducing inflammation in overweight or obese patients.

## Data Availability

The flow cytometric data used to support the findings of this study are included within the article and supplementary data file.

## Abbreviations

CCR2: Chemokine receptor 2
CFP: Circulating fibroblast precursor
DDR2: Discoidin domain receptor 2
G-CSF: Granulocyte colony-stimulating factor
GM-CSF: Granulocyte-macrophage colony-stimulating factor
HFD: High-fat diet
IFN-*γ*: Interferon-gamma
IL-: Interleukin-
MCP-1: Monocyte chemoattractant protein-1
MGAT: Mammary gland-associated adipose tissue
MFI: Mean fluorescence intensity
MHC II: Major histocompatibility complex II
MIG: Monokine induced by gamma interferon
MIP-1*α*: Macrophage inflammatory protein-1 alpha
MIP-1*β*: Macrophage inflammatory protein-1 beta
ND: Normal diet
PB: Peripheral blood
RANTES: Regulated upon activation normal T cell expressed and secreted
T2D: Type 2 diabetes
TNF-*α*: Tumor necrosis factor-alpha
VAT: Visceral adipose tissue.

## Supplementary Materials

Supplementary Materials Supplementary Table 1: comparison of high-fat diet (HFD) and normal diet (ND). Supplementary Figure 1: flow cytometric controls (unstained cells) from MGAT, VAT, and peripheral blood of ND-fed, control female C57BL/6 mice. Supplementary Figure 2: total myeloid-derived CD45^+^DDR2^+^ cells in MGAT and VAT of ND- vs. HFD-fed mice. Supplementary Figure 3: flow cytometric analysis of CD45^+^DDR2^−^ cells in MGAT, VAT, and peripheral blood of ND- vs. HFD-fed mice. Supplementary Figure 4: flow cytometric analysis of total CD3e^+^ T cells and CD3e^+^CD4^+^ T cells in MGAT, VAT, and peripheral blood of ND- vs. HFD-fed mice. Supplementary Figure 5: mediators produced by CD45^+^DDR2^+^ cells cultured in media conditioned by MGAT from HFD- or ND-fed mice.
