# Supplementary material for: High-Fat Diet Alters Immunogenic Properties of Circulating and Adipose Tissue-Associated Myeloid-Derived CD45+DDR2+ Cells
Source: Mediators Inflamm. 2019 Feb 28;2019:1648614. doi: 10.1155/2019/1648614 (PMC6421777; doi:10.1155/2019/1648614)
Supplement: Supplementary Materials — Supplementary Table 1: comparison of high-fat diet (HFD) and normal diet (ND). Supplementary Figure 1: flow cytometric controls (unstained cells) from MGAT, VAT, and peripheral blood of ND-fed, control female C57BL/6 mice. Supplementary Figure 2: total myeloid-derived CD45+DDR2+ cells in MGAT and VAT of ND- vs. HFD-fed mice. Supplementary Figure 3: flow cytometric analysis of CD45+DDR2− cells in MGAT, VAT, and peripheral blood of ND- vs. HFD-fed mice. Supplementary Figure 4: flow cytometric analysis of total CD3e+ T cells and CD3e+CD4+ T cells in MGAT, VAT, and peripheral blood of ND- vs. HFD-fed mice. Supplementary Figure 5: mediators produced by CD45+DDR2+ cells cultured in media conditioned by MGAT from HFD- or ND-fed mice. [file 1648614.f1.docx]

Supplementary Material

**High fat diet alters immunogenic properties of circulating and adipose tissue-associated myeloid-derived CD45^+^DDR2^+^ cells**

Sara J. Sidles, Ying Xiong, M. Rita I. Young, Amanda C. LaRue*

*** Correspondence:** Amanda C. LaRue: laruerc@musc.edu

| **Supplementary Table 1**. Comparison of high fat diet (HFD) and normal diet (ND) | | | |
| --- | --- | --- | --- |
| **High Fat Diet (TD.09766)** | | **Normal Diet (2018)** | |
| Nutrient | % kcal from | Nutrient | % kcal from |
| Protein | 18.3 | Protein | 24 |
| Carbohydrate | 21.4 | Carbohydrate | 58 |
| Fat | 60.3 | Fat | 18 |
| Macronutrient ingredients: casein, maltodextrin and sucrose, anhydrous milkfat and soy | | Macronutrient ingredients: wheat, corn, soy | |

**Supplementary Table 1. A comparison of the composition of high fat diet (HFD) and normal diet (ND).** HFD was composed largely of anhydrous milkfat and soy, with 60.3% kcal from fat (TD.09766, Envigo Teklad Diets). ND consisted of a standard natural ingredient chow, with 18% kcal from fat (2018 Teklad Global 18% Protein Rodent Diet, Envigo Teklad Diets).

**Supplementary Figure 1.** Flow cytometric controls (unstained cells) from MGAT, VAT and peripheral blood of ND-fed, control female C57BL/6 mice


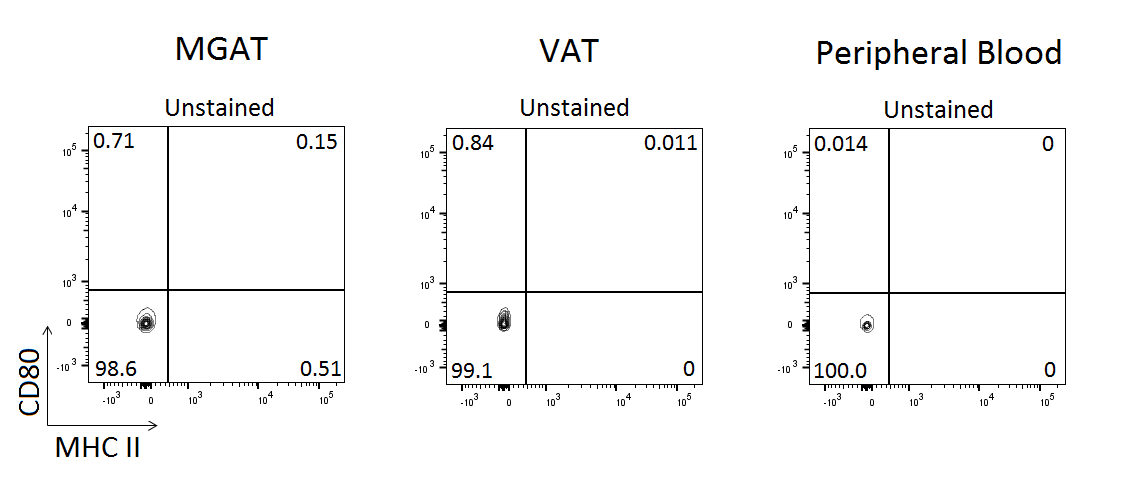


**Supplementary Figure 1. Representative flow cytometric controls (unstained cells) showing CD80 and MHC II expression in cells collected from MGAT, VAT, and peripheral blood of ND-fed, control female C57BL/6 mice.** For flow cytometric analysis, a fraction of cells isolated from mammary gland adipose tissue (MGAT), visceral adipose tissue (VAT) and peripheral blood (PB) of HFD- and ND-fed mice, respectively, were incubated without antibodies and analyzed for surface marker expression (shown in Figure 2). Shown are representative dot plots of unstained cells from MGAT, VAT and peripheral blood of ND-fed mice. Analysis of surface marker expression on unstained cells from MGAT, VAT and peripheral blood, respectively, was used to set gates for flow cytometric analysis of CD80 and MHC II expression. Total percentages of positive cells in each quadrant are shown.

**Supplementary Figure 2.** Total myeloid-derived CD45^+^DDR2^+^ cells in MGAT and VAT of ND- vs. HFD-fed mice


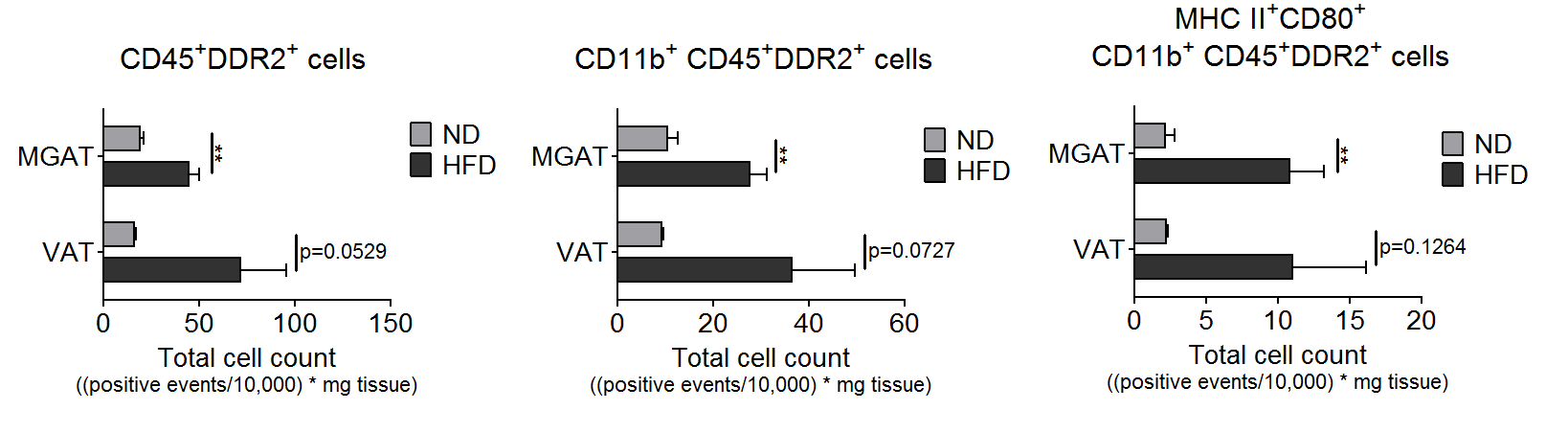


**Supplementary Figure 2. Total cell count of CD45^+^DDR2^+^ cells, CD11b^+^ CD45^+^DDR2^+^ cells, and MHC II^+^CD80^+^ CD11b^+^ CD45^+^DDR2^+^ cells in MGAT and VAT of HFD- vs. ND-fed mice.** Cell counts were calculated as ((percent positive events/10,000) * mg adipose tissue) and are presented as mean + SEM of 5 mice per group. **p<0.01

**Supplementary Figure 3.** Flow cytometric analysis of CD45^+^DDR2^-^ cells in MGAT, VAT and peripheral blood of ND- vs. HFD-fed mice


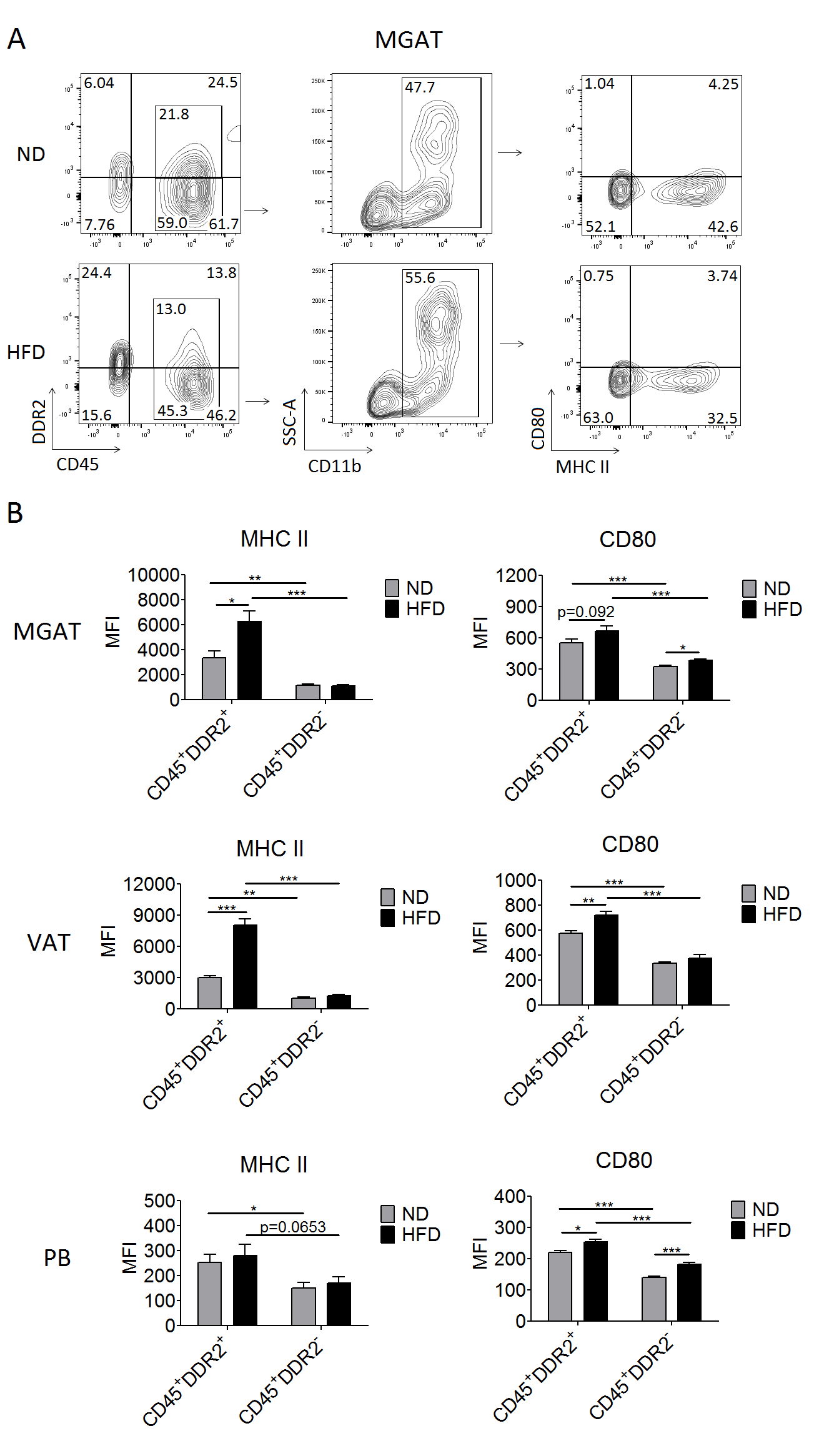


**Supplementary Figure 3. Flow cytometric analysis of myeloid-derived CD45^+^DDR2^-^ cells in MGAT, VAT, and peripheral blood of ND- vs. HFD-fed mice. (A)** Flow cytometric analysis of a representative mouse from each group, showing gating strategy and analysis of CD11b-expressing CD45^+^DDR2^-^ cells from MGAT of HFD- and ND-fed C57BL/6 mice at endpoint (9 weeks post-initiation of diet). For flow cytometric analysis, cells were first gated on single, live populations. **(B)** Graphical representation of flow cytometric analysis, showing the mean fluorescence intensity (MFI) values of MHC II and CD80 expression on CD11b-expressing, CD45^+^DDR2^-^ cells vs. CD11b-expressing, CD45^+^DDR2^+^ cells in MGAT, VAT, and peripheral blood of HFD- vs. ND fed C57BL/6 mice. Data are presented as mean + SEM of 5 mice per group. *p<0.05 **p<0.01 ***p<0.001

**Supplementary Figure 4.** Flow cytometric analysis of total CD3e^+^ T cells and CD3e^+^CD4^+^ T cells in MGAT, VAT and peripheral blood of ND- vs. HFD-fed mice


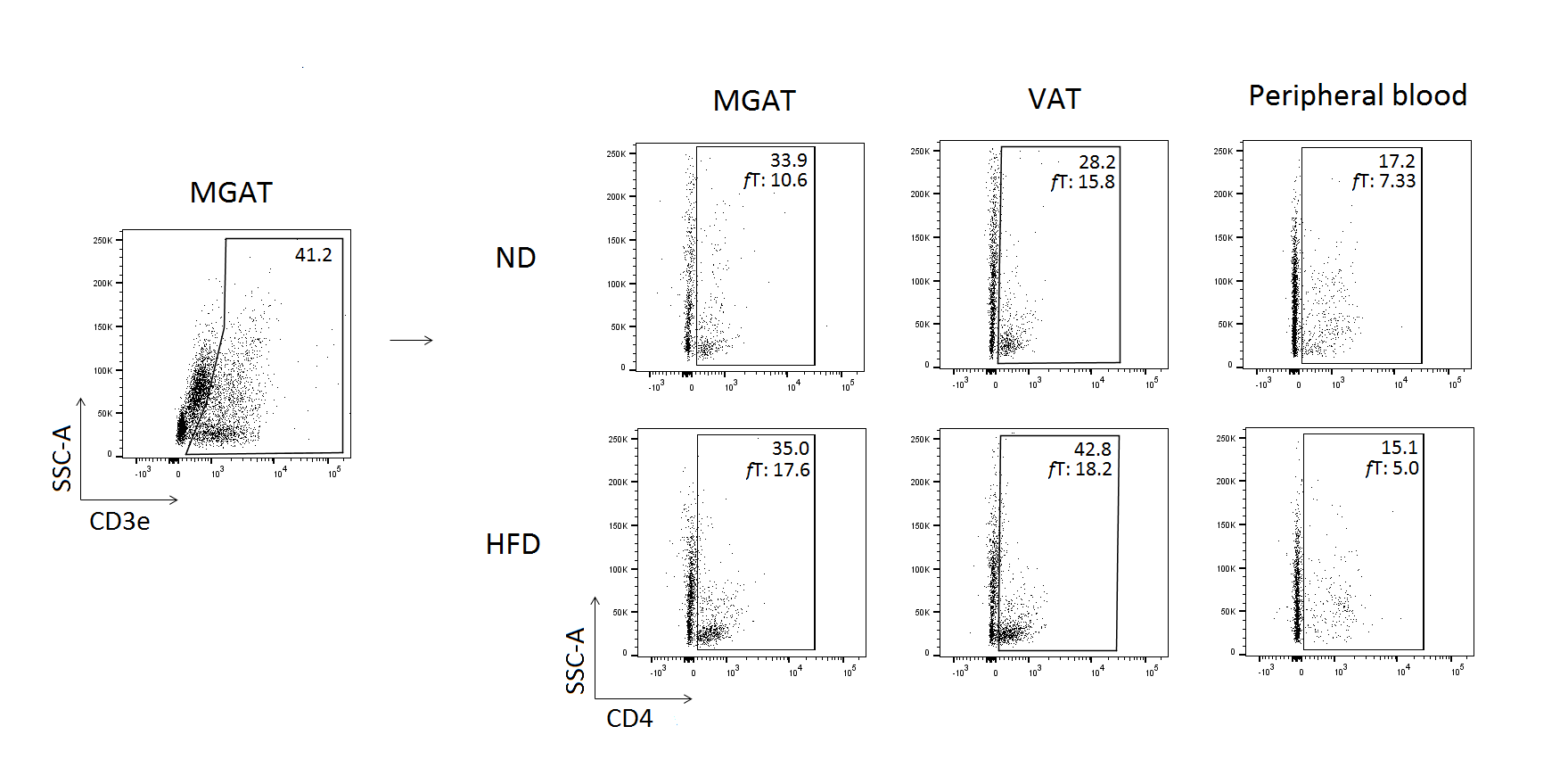


**Supplementary Figure 4. Flow cytometric gating strategy for total CD3e^+^ T cells and CD3e^+^CD4^+^ T cells in MGAT, VAT, and peripheral blood of ND- vs. HFD-fed mice.** Representative dot plots showing CD3e and CD4 surface marker expression on total live, single cell populations isolated from mammary gland adipose tissue (MGAT), visceral adipose tissue (VAT) and peripheral blood (PB) of HFD- and ND-fed C57BL/6 female mice at endpoint (9 weeks post-initiation of diet). Cells were stimulated with PMA/ionomycin cocktail + protein transport inhibitor for 5 h at 37°C prior to fixation, permeabilization and staining for surface marker and intracellular cytokine expression (shown in Figure 3). Frequency of total (*f*T) percentage was used to compare total CD3e^+^CD4^+^ T cells in ND- and HFD-fed mice. Graphical representation of mean percentages is shown in Figure 3C.

**Supplementary Figure 5.** Mediators produced by CD45^+^DDR2^+^ cells cultured in media conditioned by MGAT from HFD- or ND-fed mice

**Supplementary Figure 5. Inflammatory factors produced by CD45^+^DDR2^+^ cells cultured in the presence of media conditioned by mammary gland-associated adipose tissue (MGAT) of ND- and HFD-fed mice *in vitro*.** Sorted CD45^+^DDR2^+^ cells from peripheral blood of control, female C57BL/6 mice were cultured with media conditioned by MGAT (d1:4) for 72 hr at 37°C. Supernatants were collected for analysis via cytometric bead array. Final concentration (pg/mL) of CD45^+^DDR2^+^ cell-produced mediators was calculated by subtracting the concentration in MGAT alone from the total concentration in CD45^+^DDR2^+^ cell cultures. Data are presented as mean + SEM of triplicate wells analyzed in duplicate. *p<0.05 **p<0.01 ***p<0.001
